# Data of low-dose phase-based X-ray imaging for *in situ* soft tissue engineering assessments

**DOI:** 10.1016/j.dib.2015.12.054

**Published:** 2016-01-14

**Authors:** Zohreh Izadifar, Ali Honaramooz, Sheldon Wiebe, George Belev, Xiongbiao Chen, Dean Chapman

**Affiliations:** a Division of Biomedical Engineering, College of Engineering, University of Saskatchewan, Saskatoon, SK, Canada S7N5A9; b Department of Veterinary Biomedical Sciences, Western College of Veterinary Medicine, University of Saskatchewan, Saskatoon, SK , Canada S7N5B4; c Department of Medical Imaging, Royal University Hospital, Saskatoon, SK, Canada S7N0W8; d Canadian Light Source, Saskatoon, SK, Canada S7N2V3; e Department of Mechanical Engineering, College of Engineering, University of Saskatchewan, Saskatoon, SK, Canada S7N5A9; f Department of Anatomy and Cell Biology, College of Medicine, University of Saskatchewan, Saskatoon, SK, Canada S7N5E5

## Abstract

This article presents the data of using three phase-based X-ray imaging techniques to characterize biomaterial scaffold and soft tissues *in situ*, as reported in our study “Low-dose phase-based X-ray imaging techniques for *in situ* soft tissue engineering assessments” [1]. The examined parameters include the radiation dose, scan time, and image quality, which are all critical to longitudinal *in situ* live animal assessments. The data presented were obtained from three dimensional imaging of scaffolds *in situ* cartilage by means of synchrotron-based computed tomography-diffraction enhanced imaging (CT-DEI), analyzer based imaging (CT-ABI), and in-line phase contrast imaging (CT-PCI) at standard and low dose imaging modalities.

**Specifications Table**

**Table:** 

Subject area	Biomedical Imaging and Tissue Engineering
More specific subject area	Non-invasive scaffold and soft tissue assessment *in situ*
Type of data	Table
How data was acquired	The data was acquired based on the imaging settings, radiation dose measured by dosimeters, and image quality scoring by a clinical radiologist
Data format	In numbers from raw measurements and reconstructed CT images
Experimental factors	Imaging techniques, number of tomography projections, region of interest, and imaging resolution
Experimental features	Visualization of scaffolds and soft tissues in tissue engineering by using X-ray imaging
Data source location	Saskatoon, Canada
Data accessibility	Data are with this article

**Value of the data**

• The data provide a benchmark for comparison and selection of non-invasive phase-based X-ray imaging techniques for longitudinal live animal *in vivo* studies in tissue engineering and rheumatology research.
• The data were presented in terms of practical criteria by which one can compare the performance of different X-ray imaging techniques as well as other imaging techniques, thus allowing identifying the most appropriate techniques for given imaging applications.
• The data present the performance of different low-dose phase-based X-ray imaging techniques as applied to soft tissue engineering, which can potentially be used in other tissue engineering applications for *in vitro* and *in vivo* non-invasive assessments.

## 1 Data

The data of radiation dose, imaging scan time, and image quality scores for visualization and characterization of tissue scaffolds imaged *in situ* are given in Table 1, Table 2, Table 3 for computed tomography-diffraction enhanced imaging (CT-DEI), analyzer based imaging (CT-ABI), and extended-distance phase contrast imaging (CT-PCI) with different low-dose imaging strategies applied. Table 1 lists the data for the strategy of reducing the number of tomography projections so as to lower the radiation dose. Included in Table 1 is also the performance of the extended-distance PCI technique, which was assessed based on phase-retrieved and nonphase-retrieved PC images for comparison. Table 2 presents the data from the strategy of reduced region of interest imaging (ROI) combined with the reduced number of projections. Table 3 provides the data from the strategy of low imaging resolution combined with reduced number of projections. Table 4 lists the soft tissues and microstructural features that are visualized by using phase-based X-ray imaging techniques of CT-DEI, CT-ABI, and extended-distance CT-PCI [1]. Provided in Table 4 is also the image quality scores (IQSs) for characterization of these features by using the three phase-based imaging techniques with different low-dose imaging strategies or in their combination.

## 2 Experimental design, materials and methods

Three phase-based X-ray imaging techniques, namely diffraction enhanced imaging (DEI), analyzed-based imaging (ABI), and in-line phase contrast imaging (PCI), were investigated in our study [1] for non-invasive imaging and characterization of cartilage tissue scaffolds implanted in knee joint cartilage of piglet. To ensure the phase-based X-ray imaging techniques are suitable for longitudinal live animal imaging and tissue engineering assessments, practical aspects of these imaging techniques including imaging radiation dose and scan time are required to be modulated and optimized. To achieve this, three strategies were investigated with the phase-based X-ray imaging techniques in our study [1], and the capability and tolerance of the techniques for imaging tissue scaffolds and soft tissues at reduced radiation doses and scan times were assessed. The three low-dose strategies examined are (i) reducing the number of projections collected during CT scan, (ii) reducing the region of interest (ROI) imaged, and (iii) lowering the imaging resolution. These strategies were applied both independently and in combination to the three imaging techniques of CT-DEI, CT-ABI, and extended-distance CT-PCI.

Phase-based X-ray imaging at reduced number of CT projections was investigated by collecting, 2500 (100%), 1250 (50%), 900 (36%), 625 (25%), and 375 (15%) of two dimensional (2D) projections around the sample. Region of interest imaging (ROI) was investigated by reducing the imaging field of view in the horizontal direction from 74 mm to 43 mm, which mainly covered the scaffold implantation site. By doing so, the standard number of projections for ROI was reduced to 1800 (100%). The reduced number of CT projections for the ROI was performed by collecting 900 (50%), 600 (36%), 450 (25%), and 300 (15%) tomographic projections. Low resolution imaging was also investigated by performing CT at 37 μm (control), 74 μm and 111 μm pixel sizes. The data are also presented for 18.5 μm pixel size for extended-distance PCI to give a representation of expected dose in high imaging resolution.

The samples that were used in our study [1] were prepared by implanting 3D-printed polycaprolcatone (Mw 48,000–90,000; Aldrich, St. Louis, MO, USA) tissue scaffolds in the lateral femoral condyles of stifle joints dissected from cadaver juvenile piglets. The implanted joints with all surrounding tissues, resembling an intact joint, were imaged by using the CT-DEI, CT-ABI, and extended-distance CT-PCI (sample to detector distance of 5.9 m) at the Biomedical Imaging and Therapy Beamline-Bend magnet (BMIT-BM) of the Canadian Light Source (CLS).

To measure the delivered radiation dose, the dose rate was measured for each imaging technique by scanning the dosimeters (Luxel® by Landauer (aluminum oxide dosimeter)) across the X-ray beam in vertical direction. The dose rate was then obtained from the measured total dose by

$$\dot{D}=\frac{V}{h}D$$

where $\dot{D}$ is the rate of radiation dose, *V* is the vertical scanning speed, *h* is the beam height measured at the sample location, and *D* is the total radiation dose measured by the dosimeter. The vertical scanning speeds (*V*) set for DEI/ABI and PCI were 0.1 mm/s and 0.2 mm/s, respectively. The beam height was measured on Linagraph Direct Print papers (Kodak, Type 2167 standard). The total delivered dose to the samples during imaging was then calculated according to

$$D=\dot{D}\times \Delta t$$

where $\dot{D}$ is the calculated dose rate and Δt is the total exposure time. The total radiation dose data in the tables are given in effective dose equivalent in a unit of Sievert (Sv). The associated dose conversion from the measured dose in Gray (Gy) to effective dose is performed according to

$$D(\mathrm{Sv})=D(\mathrm{Gy})\times W_{R}\times W_{T}$$

where *D* is absorbed dose, *W*_*T*_ is a tissue radio-sensitivity weighting factor (0.01 for cartilage [2]), and *W*_*R*_ is a radiation weighting factor (1 for photon particle [2]). The imaging scan time was calculated based on the exposure time given for collecting single 2D projection multiplied by the total number of 2D projections collected in each CT scan.

The image quality scores were obtained from observer image quality assessments. A 5-point scoring system was used by a clinical radiologist to characterize the image quality in terms of visualizing tissue scaffold, soft tissues, microstructural, and anatomical features in images obtained from different imaging techniques and low dose imaging strategies. In the employed image scoring system, 5 represents “best”, 4 “optimal, 3 “adequate, 2 “insufficient” and 1 “poor” image quality.

## Figures and Tables

**Table 1:** Total effective dose, scan time, and observer image quality score (IQS) for visualizing scaffolds *in situ* with the strategy of reduced number of projections in CT imaging.

% Proj	CT-DEI			CT-ABI			Extended-distance CT-PCI			
	Dose^a^	Scan time^b^	IQS	Dose^a^	Scan time^b^	IQS	Dose^a^	Scan time^b^	IQS	IQS (NP^c^)
100	34.5	294.6	5	17.2	147.3	4	30.1	7.8	4	3
50	17.2	147.3	4	8.6	73.7	4	15.1	3.9	4	2
36	12.4	106.1	4	6.2	53.1	4	10.8	2.8	4	2
25	8.6	73.7	4	4.3	36.9	4	7.5	2.0	3	1
15	5.2	44.3	3	2.6	22.1	3	4.5	1.2	3	1

a Total effective dose (mSv).

b Total scan time (min).

c Nonphase-retrieved.

**Table 2:** Total effective dose, scan time, and observer image quality score (IQS) for visualizing scaffolds *in situ* with the strategy of reduced region of interest (ROI) combined with reduced number of projections in CT imaging.

% Proj	CT-DEI			CT-ABI			Extended-distance CT-PCI		
Dose^a^	Scan time^b^	IQS	Dose	Scan time	IQS	Dose	Scan time	IQS
100	24.8	212.1	4	12.4	106.0	4	19.4	5.0	4
50	11.5	98.3	4	6.2	53.1	4	9.5	2.5	4
36	8.6	73.7	4	4.3	36.9	4	6.3	1.6	3
25	6.9	59.0	4	3.1	26.6	3	4.8	1.2	3
15	4.1	35.4	3	2.1	17.7	2	3.2	0.8	3

a Total effective dose (mSv).

b Total scan time (min).

**Table 3:** Total effective dose, scan time, and observer image quality score (IQS) for visualizing scaffolds *in situ* with the strategy of low imaging resolution combined with reduced number of projections in CT imaging.

**CT-DEI (%)**	37 µm				74 µm			111 µm					
Dose^a^	Scan time^b^		IQS	Dose	Scan time	IQS	Dose	Scan time	IQS			
100%	34.5	294.6		5	8.8	75.3	4	4.0	34.4	4			
50%	17.2	147.3		4	4.4	37.7	4	2.0	17.2	3			
36%	12.4	106.1		4	3.2	27.1	4	1.4	12.4	3			
25%	8.6	73.7		4	2.2	18.8	3	1.0	8.6	3			
15%	5.2	44.3		3	1.3	11.3	2	0.6	5.2	2			

**CT-ABI (%)**	37 µm				74 µm			111 µm					
Dose	Scan time	IQS		Dose	Scan time	IQS	Dose	Scan time	IQS			

100	17.2	147.3	4		4.4	37.7	4	2.0	17.2	4			
50	8.6	73.7	4		2.2	18.8	4	1.0	8.6	4			
36	6.2	53.1	4		1.6	13.6	4	0.7	6.2	3			
25	4.3	36.9	4		1.1	9.4	3	0.5	4.3	3			
15	2.6	22.1	3		0.7	5.7	2	0.3	2.6	2			

**Ext.-dist. CT-PCI (%)**	18.5 µm				37 µm			74 µm			111 µm		
ose	Scan time	IQS		Dose	Scan time	IQS	Dose	Scan time	IQS	Dose	Scan time	IQS

100	187.0	48.5	5		30.1	7.8	5	9.6	2.5	4	3.4	0.9	3
50					15.1	3.9	4	5.8	1.5	3	1.5	0.4	3
36					10.8	2.8	4	3.5	0.9	3	1.2	0.3	2
25					7.5	2.0	3	2.4	0.6	2	0.8	0.2	1
15					4.5	1.2	3	1.4	0.4	2	0.5	0.1	1

a Total effective dose (mSv).

b Total scan time (min).

**Table 4:** Image quality scoring for *in situ* visualization of soft microstructural features captured by the phase-based CT imaging with low-dose imaging strategies or in their combination.

**Microstructural features**	Cartilage defect perimeters	Cartilage anatomy/distinct interfaces	In-cartilage vascular channels	Fat tissue perimeters & internal structure	Ligament anatomy & internal structure	Muscle tissue perimeters	Distortion of tissue parenchyma	Surgery suture	Covering periosteum patch
CT-DEI (37 µm) (%)									
100	5	5	5	4	5	5	5	5	5
50	4	4	4	4	4	4	4	4	4
36	4	4	4	4	4	4	4	4	4
25	4	4	4	4	4	4	4	4	4
15	3	3	3	3	3	3	3	3	3
ROI CT-DEI (37 µm) (%)									
100	4	4	4	4	4	4	4	4	4
50	4	4	4	4	4	4	4	4	4
36	4	4	4	4	4	4	4	4	4
25	4	4	3	3	4	4	4	4	4
15	3	3	3	3	3	3	3	3	3
CT-DEI (74 µm) (%)									
100	4	4	4	4	4	4	4	4	4
50	4	4	4	3	4	4	4	4	3
36	3	4	4	3	4	3	3	3	2
25	2	3	3	3	3	3	2	3	1
15	2	3	2	3	3	2	1	3	1
CT-DEI (111 µm) (%)									
100	3	4	4	3	4	4	4	4	4
50	3	4	3	3	3	3	3	3	3
36	3	3	2	3	3	3	3	3	2
25	2	3	2	3	2	2	2	2	1
15	2	2	1	2	2	2	2	2	1
CT-ABI (37 µm) (%)									
100	4	5	4	4	5	4	4	5	4
50	4	4	4	4	4	4	4	4	4
36	4	4	4	3	4	4	4	4	4
25	4	4	4	3	4	4	4	4	3
15	3	3	3	2	3	3	3	3	3
ROI CT-ABI (37 µm) (%)									
100	4	4	4	3	4	4	4	4	4
50	3	3	3	3	4	3	3	4	3
36	3	3	3	3	3	3	3	4	3
25	3	3	3	3	3	3	3	3	3
15	2	2	2	2	2	2	2	2	2
CT-ABI (74 µm) (%)									
100	4	4	4	4	4	3	3	4	4
50	4	3	3	3	3	3	3	4	4
36	3	3	3	3	3	3	3	3	3
25	2	3	2	2	2	2	2	3	2
15	2	1	1	1	1	1	1	2	2
CT-ABI (111 µm) (%)									
100	4	4	3	3	3	3	3	4	4
50	4	3	3	3	3	3	3	4	4
36	3	3	2	3	2	2	2	3	2
25	2	2	2	2	2	2	2	3	2
15	2	1	1	1	1	1	1	2	2
Extended-distance CT-PCI (37 µm) (%)									
100	5	4	5	5	4	4	4	4	4
50	4	4	4	4	4	4	4	3	3
36	4	3	3	4	3	3	3	2	2
25	3	2	2	3	2	2	2	2	2
15	2	1	1	2	2	2	2	1	1
ROI Extended-distance CT-PCI (37 µm) (%)									
100	4	3	3	4	4	3	4	4	3
50	4	3	3	4	3	3	4	3	3
36	3	2	2	4	3	2	3	2	2
25	3	2	2	3	2	2	3	2	2
15	2	1	1	2	2	2	2	1	1
Extended-distance CT-PCI (74 µm) (%)									
100	4	3	3	4	3	3	3	3	3
50	3	3	2	3	3	2	3	3	2
36	3	2	2	3	3	2	3	2	2
25	3	2	1	2	2	2	2	2	2
15	2	1	1	2	2	2	2	1	1
Extended-distance CT-PCI (111 µm) (%)									
100	3	3	2	4	3	3	3	3	3
50	3	3	2	3	3	2	3	3	2
36	2	2	2	2	3	2	2	2	2
25	1	1	1	2	2	2	2	2	2
15	1	1	1	1	1	1	1	1	1
